# Safety considerations for assessing the quality of apps used during pregnancy: A scoping review

**DOI:** 10.1177/20552076231198683

**Published:** 2023-09-04

**Authors:** Alayna Carrandi, Melanie Hayman, Cheryce L Harrison

**Affiliations:** 1 Monash Centre for Health Research and Implementation, 2541 Monash University, Australia; 2 Medical and Applied Sciences, 6939 CQUniversity, Australia; 3 Monash Centre for Health Research and Implementation, 2541 Monash University, Australia; 4 Diabetes and Vascular Medicine, 2538 Monash Health, Australia

**Keywords:** mHealth, digital health, apps, pregnancy, healthy lifestyle

## Abstract

**Objective:**

Pregnant women are increasingly turning to apps targeting knowledge and behaviour change for supporting healthy lifestyles and managing medical conditions. Yet, there is growing concern over the credibility and safety of content within mobile health (mHealth) apps. This scoping review aimed to systematically and thematically consolidate safety considerations described in reviews evaluating pregnancy-specific apps.

**Methods:**

PubMed, Ovid MEDLINE® and EPub, CINAHL, Web of Science, Cochrane Libraries, and SCOPUS were systematically searched to identify reviews that assessed apps targeting pregnant women. Data related to safety were extracted and thematically analysed to establish a set of relevant safety considerations.

**Results:**

Sixteen reviews met the inclusion criteria. The included reviews assessed an average of 27 apps each and targeted pregnancy topics, such as nutrition and physical activity. Five major and 20 minor themes were identified, including information, transparency, credibility, privacy and security, and app tailoring. Information, transparency, and credibility relate to the evidence base of information within the app, privacy and security of apps relate to the protection of personal information and data, and app tailoring relates to the consideration of contextual factors, such as local guidelines and digital health literacy.

**Conclusions:**

Results present possible safety considerations when evaluating pregnancy-specific apps and emphasise a clear need for consumer guidance on how to make informed decisions around engagement and use of mHealth apps during pregnancy.

## Introduction

Globally, more than 6 billion people own a mobile device, equating to over 80% of the population in most countries worldwide.^1^ Developed countries are shown to have higher rates of smartphone ownership compared with major developing countries (76% vs 46%),^2^ with ownership demographically highest in those who are younger (under 35 years old), educated, and earning a higher income.^2^ Rapid innovations in smartphone technologies, including growth in global mobile cellular network coverage, availability of downloadable applications, and continued market penetration, have each paved the way for advancements in mHealth. mHealth is defined broadly as medical and public health practice supported by mobile devices and wireless technologies.^3^ A significant driver of mHealth is the exponential increase in the availability and use of smartphone applications (apps) and wearable devices which present as potentially powerful interactive tools for consumers to manage their health information,^4^ with lifestyle, fitness, and weight management apps and tracking devices the most popular in the market.^5^

Apps are particularly popular during pregnancy and have become an important source of information for women seeking health-related guidance and support,^6^ rapidly replacing traditional methods of obtaining information including from health professionals and written resources.^7–9^ An Australian cross-sectional survey of 410 pregnant women reported ~75% of respondents used at least one app during pregnancy,^9^ with comparable findings reported elsewhere.^7^ Pregnant women are increasingly turning to apps targeting knowledge and behaviour change related to pregnancy, such as lifestyle (diet and/or physical activity), maternal awareness of decreased foetal movements, maternal weight monitoring, and breastfeeding.^10^ It is, therefore, not surprising that there are currently more mHealth apps available for pregnancy than for any other medical topic,^11^ with apps for pregnancy, childbirth, and child care some of the most common health apps used by women broadly.^7^

Whilst apps designed for use in pregnancy are prolific and are ideally placed to provide accessible information and support to women, there is growing concern over their credibility and safety of content, lack of regulation, and ability to influence behaviour change and self-management.^10,12^ This includes several recent reviews highlighting inaccurate content, lack of appropriate screening processes, lack of expert involvement, poor or unclear evidence base of content, and poor validation.^6,13–20^ This may increase the risk of misinformation and subsequent consequences including injury, complications, and/or adverse pregnancy outcomes for both the mother and child.^17^ Whilst a variety of tools and frameworks, including the Mobile Application Rating Scale (MARS);^21^ Coventry, Aberdeen, and London-Refined (CALO-RE) framework;^22^ and APPLICATIONS (app comprehensiveness, price, privacy, literature used, in-app purchases, connectivity, advertisements, text search field, images/videos, other special features, navigation ease, and subjective presentation) scoring system,^23^ are available to evaluate mHealth apps, they predominantly assess engagement, functionality, aesthetics, and information quality. As such, evidence-based tools to robustly evaluate the safety considerations of mHealth apps are lacking. Thus, we aimed to systematically and thematically consolidate safety criteria, considerations, and concerns described in the assessments of apps targeting pregnant women.

## Methods

We used the five-stage approach described by Arksey and O’Malley^24^ and followed the PRISMA ScR (Preferred Reporting Items for Systematic Reviews and Meta-Analyses extension for Scoping Reviews) checklist^25^ to guide the reporting of this review (see Appendix 1). Since this review was a scoping review rather than a systematic review, it did not fit the current PRISMA criteria to register with PROSPERO.

### Stage 1: identify the research question(s)

Following a preliminary literature search and discussions between the author group, the following research questions were developed to reflect the population, context, and content of the review:

► Which assessment tools are currently being used to assess commercially available apps for women that target lifestyle or medical conditions during pregnancy?
► What safety-related considerations are reported in assessments of commercially available apps for pregnant women?

### Stage 2: identifying relevant studies and search strategy

A systematic search strategy was used to identify peer-reviewed studies published in English between January 2011 and April 2022 reporting on the assessment of apps for pregnant women. An initial search was conducted on 24 November 2021 and updated on 28 April 2022, using the following key search terms: mobile health app; review, evaluation, or assessment; and pregnancy. The following databases were searched: PubMed, Ovid MEDLINE® and EPub, CINAHL, Web of Science, Cochrane Libraries, and SCOPUS. The complete search strategies for each database are detailed in Appendix 2.

Study designs were limited to reviews (e.g. content analyses, content evaluations, and systematic or scoping reviews) that assessed apps for pregnancy regarding any outcome of interest (e.g. quality, credibility, safety, privacy, and functionality). Reviews had to identify and assess at least two commercially available apps, defined as apps that are publicly available to consumers with mobile phones (e.g. accessible via the iTunes app store or Google Play Store), targeting lifestyle behaviours (e.g. nutrition or physical activity) or any associated medical conditions (e.g. mental health, pregnancy monitoring, gestational diabetes mellitus, or preeclampsia) during pregnancy. Reviews were excluded if they were not a review of commercially available apps (number of apps < 2) or if they only targeted other population groups (i.e. not pregnant women). Apps targeting multiple life stages (e.g. preconception and postpartum) were included provided they were designed for use during pregnancy also. Reviews were further excluded if they strictly evaluated the effectiveness, efficacy, or feasibility of apps. Only reviews published in English were included in the review.

### Stage 3: review selection

Following the search, all identified reviews were uploaded into Covidence^26^ and duplicates removed. Two independent reviewers (AC and MH) screened titles and abstracts against the inclusion and exclusion criteria, retaining remaining records for full-text review. Reasons for exclusion at the full-text stage were recorded. Any disagreements that arose between the reviewers at each stage of the screening process were resolved by a third reviewer (CLH).

### Stage 4: charting the data

The authors (AC, MH and CLH) designed a data extraction form using other seminal reviews in different content areas^17,^^27–30^ (Appendix 3). We developed an extensive list of safety considerations from the findings of these reviews. Safety considerations included the following: inclusion of disclosures, content and information validity, expertise of app developers’ including credentials and/or qualifications, and stakeholder consultation with relevant groups (e.g. obstetricians, midwives, or allied health professionals). All data related to the aforementioned safety considerations were extracted verbatim from the included reviews. The data extraction tool also included details about the health domain of the apps being assessed (e.g. lifestyle behaviours or medical condition), aim(s) of the review, how apps were identified (e.g. database and platforms searched), tools used to assess the apps and their respective outcomes, and number of apps included in the review. The data extraction form was piloted independently by three authors (AC, MH and CLH) for a randomised selection of five included reviews. The research team met to discuss any additions or changes necessary before progressing with the data extraction process. The final data extraction form is presented in Appendix 3. Data from all included reviews were independently extracted by two reviewers (AC and MH). Any disagreements that arose between the reviewers were resolved by a third reviewer (CLH).

### Stage 5: collating, summarising and reporting the results

Review characteristics were presented in summary tables, and data related to safety were thematically analysed using the approach set out by Braun and Clarke.^31^ Briefly, the six phases are as follows: (a) familiarising yourself with the data; (b) developing initial codes that capture important features of the data relevant to the research question; (c) generating initial themes; (d) developing and reviewing themes; (e) refining, defining, and naming themes; and (f) writing up the themes and contextualising them in relation to the research question. Two members of the research team (AC and MH) reviewed the data then met to discuss and develop an initial set of codes. Themes were generated from the initial set of codes independently by one team member (AC) and reviewed independently by another team member (MH) to achieve consensus. Two reviewers (AC and MH) then met to ascertain major and minor themes, by categorising data to develop an extensive list of potential safety considerations when assessing pregnancy apps. Major themes captured broad safety features described in the data, and minor themes captured more specific features.

## Results

**Figure 1.**
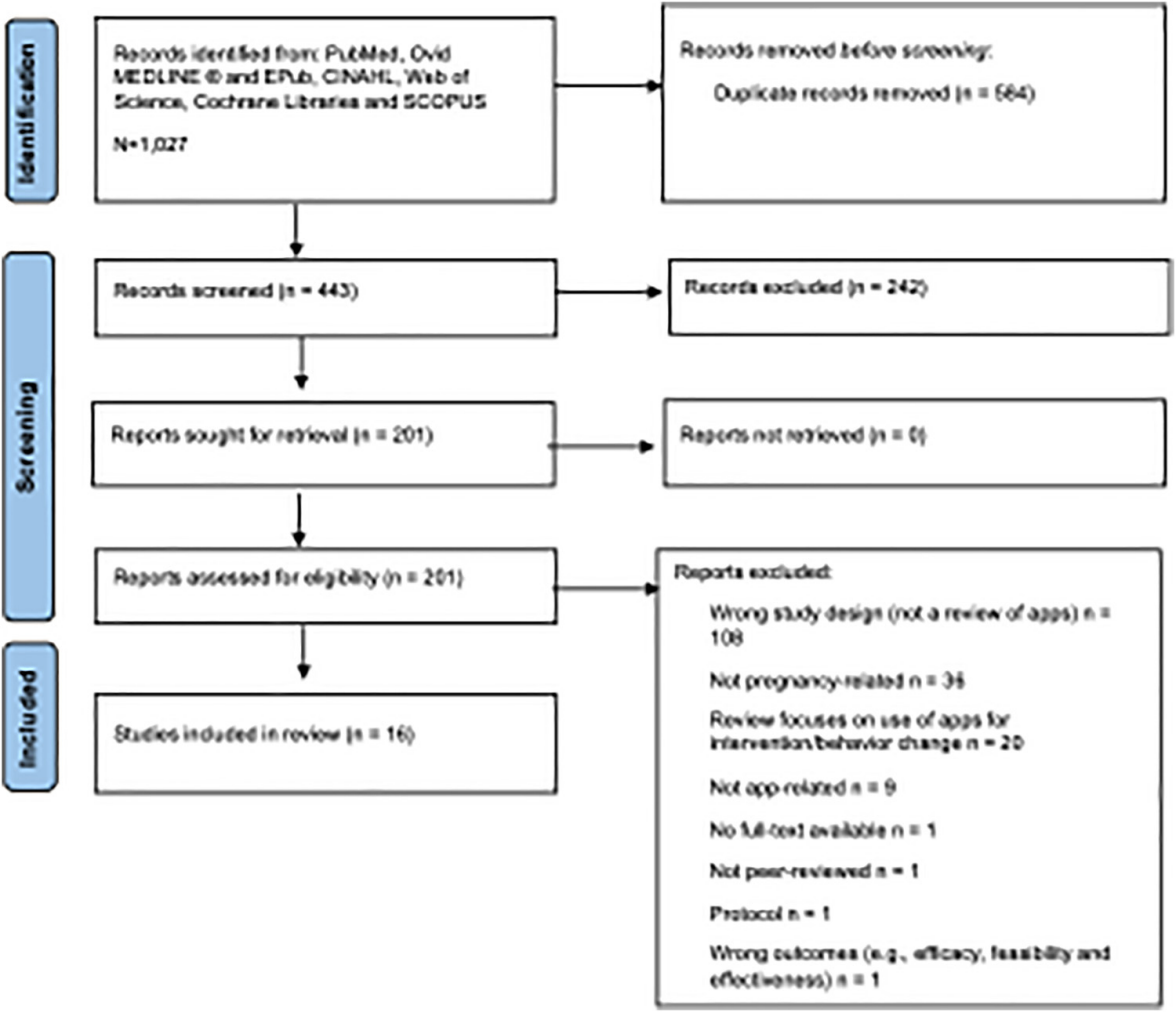
Preferred reporting items for systematic reviews and meta- analyses extension for scoping reviews flow chart.

The search retrieved a total of 1027 reviews, of which 584 duplicates were removed. After an initial screening of the remaining 443 abstracts and titles, 242 reviews that did not meet the eligibility criteria were excluded, and 201 were selected for full-text screening. After the full-text review, 16 reviews remained that fulfilled the inclusion criteria for this scoping review (Figure 1).

### Review characteristics

Included reviews were published between 2015 and 2022 (Table 1). Three-quarters (*n* = 12) of reviews searched the iTunes app store and Google Play Store for pregnancy-related apps. Other databases used to identify apps were websites and academic literature databases.

**Table 1.** Characteristics and assessment outcomes of identified reviews of pregnancy apps (*n* = 16).

First Author (Year of Publication)	Aim(s)	Apps Included in Review (*N*)	Health Domain	Platforms Searched	Assessment Tool			No. of People Involved in Extraction
					Name	Validated (Yes/No)	Quantitative Score (If Available)	
Brown et al. (2019)^18^	Assess the quality of pregnancy apps and whether they included BCT and/or pregnancy-specific nutrition information	51	Nutrition	1.iTunes (Aus) 2.Website: https://fnd.io/	MARS	Yes	Mean quality score: 3.05/5.00	1
					CALO-RE	Yes	Median number of BCTs per app: 3	1
					Content analysis	No	Median number of nutrition information topics per app: 3	NR
Bland et al. (2020)^32^	Evaluate the coverage and content of nutrition information in apps available to UK pregnant women	29	Nutrition	1.iTunes (UK) 2.GooglePlay	Silberg's standards	No	Mean quality score: 3.1/8	NR
					Content analysis	No	-	2
					BCT taxonomy	No	Mean number of BCTs per app: 10	NR
Bachiri et al. (2016)^33^	Analyse the functionalities of apps specific to pregnancy monitoring	33	Pregnancy monitoring	1.iTunes 2.GooglePlay	Quality assessment questionnaire	No	Mean quality score: 46.24% (∼16/35)	2
Muñoz-Mancisidor et al. (2021)^34^	Identify the apps available in Spanish that can be recommended to pregnant women based on their content, BCTs, and quality	25	Pregnancy monitoring	1.iTunes 2.GooglePlay	MARS	Yes	Median objective quality score: 2.94/5 Median subjective quality score: 1.75/5	2
					Content analysis	No	Median pregnancy topics identified: 23	1
					BCT taxonomy	Yes	Mean number of BCTs identified: 2	2
O’Donnell et al. (2016)^35^	Evaluate the content of two free, pregnancy-specific apps and their accuracy and adherence to prenatal care guidelines	2	General pregnancy monitoring and education	Nil—two apps targeted for purposes of this study	Content analysis	No	-	2
Wu et al. (2021)^36^	Identify, evaluate, and summarise apps that contain prenatal genetic testing information	64	Prenatal genetic testing	1.iTunes 2.GooglePlay	AQASS	No	Mean quality score: 13.5/27	2
Tassone et al. (2020)^37^	Examine how well apps for diet tracking and prevention of gestational diabetes met the needs of target users	17	Gestational diabetes mellitus	1.iTunes (USA) 2.GooglePlay	Framework developed by authors	No	Mean quality score: 11.8/31	2
de Sousa Gomes et al. (2019)^38^	Evaluate apps about preeclampsia for the health promotion of pregnant women	11	Preeclampsia	1.iTunes 2.GooglePlay	Synoptic framework	No	Mean quality score: 18/30	NR
Tsai et al. (2022)^39^	Assess the quality of apps targeting perinatal depression and/or anxiety	37	Perinatal depression and anxiety	1.iTunes (USA, UK, Can, and Aus) 2.GooglePlay (USA, UK, Can, and Aus)	MARS	Yes	Mean quality score: 3.6/5	2
Daly et al. (2019)^40^	Explore information about decreased foetal movement provided through apps intended for use during pregnancy	24	Foetal movement	1.GooglePlay	Content analysis	No	-	NR
Musgrave et al. (2020)^10^	Identify and assess the quality of the top pregnancy apps in Australia	10	General pregnancy monitoring and education	1.iTunes 2.GooglePlay	MARS	Yes	Mean quality score in 2017: 3.01/5 Mean quality score in 2019: 3.40/5	2
					CALO-RE	Yes	Mean number of BCTs for breastfeeding: 5 Mean number of BCTs for pregnancy weight: 4 Mean number of BCTs for maternal awareness of foetal movements: 5	2
Womack et al. (2020)^41^	Examine specific risk recommendations made by popular pregnancy-tracking apps	48	General pregnancy monitoring and education	1.iTunes 2.GooglePlay	SARF	No	Mean number of pregnancy topics addressed: 3.85	1
Hayman et al. (2021)^42^	Assess app quality, features and the presence of BCTs in apps designed to promote physical activity among pregnant women	19	Physical activity	1.iTunes (Aus) 2.GooglePlay	MARS	Yes	Mean quality score: 3.5/5	1
					BCT taxonomy	No	Mean number of BCTs per app: 4.74	2
Frid et al. (2021)^43^	Identify pregnancy mobile apps and evaluate the quality of apps	29	General pregnancy monitoring and education	Web search engine developed by Google	Modified APPLICATIONS	No	Mean quality score: 9.4/16	3
Hayman et al. (2022)^17^	Examine content of apps that promote physical activity and exercise in pregnancy to assess the alignment with current recommendations	27	Physical activity	1.iTunes (Aus) 2.GooglePlay Store	Content analysis	No	-	2
Evans et al. (2022)^44^	Evaluate a methodology for searching and reviewing apps that support pregnant women with symptoms of anxiety	12	Anxiety	1.iTunes (UK) 2.GooglePlay 3.Website search	Content analysis	No	-	2

AQASS: app quality assessment scoring system; Aus: Australia; BCT: behaviour change techniques; CALO-RE: Coventry, Aberdeen, and London-Refined; Can: Canada; MARS: Mobile Application Rating Scale; NR: not reported; SARF: social amplification and attenuation of risk framework; UK: United Kingdom; USA: United States of America.

Almost half (*n* = 6) of the included reviews assessed general pregnancy-monitoring and education apps,^10,33–35,41,43^ whilst two reviews assessed apps focused on nutrition,^18,32^ two evaluated physical activity apps,^17,^^42^ two evaluated mental health apps,^39,44^ one evaluated apps providing guidance on decreased foetal movement,^40^ one evaluated gestational diabetes mellitus prevention apps,^37^ one evaluated preeclampsia apps,^38^ and one appraised apps containing information on prenatal genetic testing.^36^ The included reviews assessed an average of 27 apps (range 2–64) each, comprising a total of 445 apps included in this review.

Overall, 50% (*n* = 8) of the included reviews used more than one assessment tool to appraise the apps’ functionality, quality, and inclusion of behaviour change techniques (BCTs). Of the 11 assessment tools identified, two were validated, including the MARS and CALO-RE frameworks. Three reviews assessed the functionality of apps. Of these reviews, one used a modified APPLICATIONS scoring system,^43^ one used an adapted synoptic framework,^38^ and one used a quality assessment questionnaire developed by a midwife and gynaecologist.^33^ Twelve reviews assessed the quality of apps, the inclusion of BCTs, and pregnancy-specific information. Three of these reviews used more than one assessment tool to achieve this aim.^10,18,34^ Five reviews used MARS,^10,18,34,39,42^ one used the app quality assessment scoring system (AQASS),^36^ three used dichotomous BCT taxonomies,^32,34,42^ and two used the CALO-RE framework.^10,18^ Two reviews used a tool developed by the authorship group to identify the presence of various functionalities contained within the apps.^33,37^ Seven reviews used content analyses to evaluate the accuracy of the content with respect to national recommendations or clinical guidelines.^17,^^18,32,34,35,40,44^ One review examined the risk recommendations made by apps and their associated evidence using the social amplification and attenuation of risk framework (SARF).^41^ One review assessed the apps’ accountability—authors credited, authors’ affiliation, sponsorship disclosure, whether the app had been modified recently, and the provision of information sources or references.^32^ One review assessed the information validity and relevance to target users using a structured framework for app characterisation.^37^

Assessment tools, such as MARS, AQASS, and other frameworks developed by the authorship group, were used to report on the quality of apps. The overall reported quality scores showed wide variability across the health domains. The mean quality score for apps related to prenatal genetic testing was 50.00%^36^; gestational diabetes, 38.06%^37^; preeclampsia, 60.00%^38^; mental health, 72.00%^39^; and physical activity, 70.00%.^42^ The mean overall quality scores reported for nutrition apps were generally lower at 38.75%^32^ and 61.00%.^18^ Mean overall quality score for pregnancy-monitoring apps was 46.24%,^33^ median objective quality score was 58.80%,^34^ and median subjective quality score was 35.00%.^34^ Mean overall quality scores for general pregnancy-monitoring and education apps ranged from 58.75%^43^ to 68.00%.^10^

Assessment tools, such as CALO-RE and BCT taxonomies, were used to report on the presence of BCTs in apps. The average number of BCTs in apps focused on nutrition was 10,^32^ and the median number of BCTs was 3.^18^ The mean number of BCTs in pregnancy-monitoring apps was 2, and the median number of topics related to pregnancy monitoring identified in the apps was 23.^34^ The average number of BCTs included in apps targeting physical activity was 4.74.^42^ The mean number of BCTs identified in pregnancy-monitoring and education apps for breastfeeding was 5; for pregnancy weight, 4; and for maternal awareness of foetal movements, 5.^10^ The average number of pregnancy topics identified in general pregnancy-monitoring and education apps was 3.85.^41^

### Thematic analysis of safety

The process of inductive coding generated 20 minor themes related to safety which were grouped into five major themes including information, transparency, credibility, privacy and security and, app tailoring and responsiveness (Table 2). Two studies did not refer to aspects of safety in their app review.^33,38^

**Table 2.** Thematic analysis of safety consideration identified in reviews of pregnancy app (*N* = 16).

Major Themes	Minor themes	Description
Information	a. Evidence-based information (*n* = 10)	Use of information from established guidelines^10,17,18,36,37,39–41,43,44^
	b. Inclusion of citations for health-related information (*n* = 7)	Inclusion of references and/or citations to support the health information provided within the app^10,32,36,38,40,41,44^
	c. Ambiguous or inconsistent information (*n* = 3)	Missing, incomplete, or inconsistent information^10,35,41^
	d. Comprehensiveness of information (*n* = 4)	Superficial provision of information^17,^^37,38,43^
	e. Guidance for self-care or self-assessment (*n* = 3)	Instruction to count kicks manually, product recommendation for self-care (e.g. foetal Doppler ultrasound), and self-assessment^10,40,43^
	f. Harmful or inaccurate information (*n* = 4)	Information contradictory to current evidence-based guidelines/recommendations^10,17,18,40^
	g. Safety messaging (*n* = 5)	Messaging encouraging women seek medical advice if concerned, in event of an emergency^10,33,38,43,44^
Transparency	a. Affiliations, ownership, sources of funding and endorsement (*n* = 6)	Disclosure of sponsorships, affiliations, ownership, sources of funding and endorsements within the app^18,32,36,38,40,44^
	b. Disclaimer or terms and conditions (*n* = 1)	Disclaimers and/or terms and conditions within the app^17^
Credibility	a. Recommended by a health provider (*n* = 1)	A health provider recommended the use of the app during pregnancy^37^
	b. Developed in consultation with a health provider (*n* = 2)	Developed in consultation with medical institution, clinicians or health researchers^38,44^
	c. Tested or trialled (*n* = 2)	Tested by research studies to determine quality and efficacy^36,44^
Privacy & Security	a. Data and password protection (*n* = 2)	Data protection and the use of password protection within the app^33,43^
App Tailoring & Responsiveness	a. Sync with medical equipment or plug-in (*n* = 2)	Ability to connect to sensors for pregnancy monitoring^33,40^
	b. Customisation (*n* = 5)	Account for pre-existing medical conditions, tailoring of information based on user's health status and location-specific information^17,^^18,35–37^
	c. Accessibility (*n* = 2)	Reading level, visual information, and translated information^36,44^
	d. Safety alerts and notifications (*n* = 4)	Ability to alert or call to action in response to user data^10,38,40,43^
	e. Tracking ability (*n* = 5)	Capability to track, monitor behaviours, log, follow, manage, record, register, report, or count health information or data^10,37,38,40,43^
	f. Requiring medical clearance (*n* = 1)	Advised user to seek medical clearance or specifically asked whether the user had obtained approval or clearance from their healthcare provider prior to engaging in exercise^17^

Within the major theme information, the following seven minor themes were identified: evidence-based information, citations for health-related information, ambiguous or inconsistent information, comprehensiveness of information, guidance for self-care or self-assessment, harmful or inaccurate information, and safety messaging. Ten reviews assessed the presence of evidence-based information by whether the content within the apps aligned with national recommendations or clinical guidelines.^10,17,18,36,37,39–41,43,44^ Seven reviews evaluated whether the apps within their review included references to support health-related information within the apps.^10,32,36,38,40,41,44^ Reliability of information was determined through the use of references and citations and the credibility of those information sources. For example, credible information sources may include medical practice or medical research groups, named clinicians or health professionals, peer-reviewed articles, governmental organisations, and authoritative professional associations.^36,41^ Three reviews identified incomplete, ambiguous, or inconsistent information^10,35,41^ within the apps that were reviewed. Information was deemed incomplete, ambiguous, or inconsistent when the apps were missing content or falling short of providing useful information aligned with national recommendations or clinical guidelines.^10,41^ Four reviews deemed the comprehensiveness of information an important consideration when assessing apps.^17,^^37,38,43^ Information was considered comprehensive if it included information related to the signs, symptoms, diagnosis, prevention, epidemiology, and complications associated with the health topic.^38^ Three reviews assessed whether the apps within their review contained self-care or self-assessment guidance.^10,40,43^ Guidance for self-care may include the option for users to set reminders to participate in self-care activities, such as stimulating foetal movement through consumption of food or drink,^40^ or health promotion education components, such as monitoring salt intake, walking, and practicing yoga.^38,43^ Four reviews evaluated whether the apps within their review presented harmful or inaccurate information.^10,17,18,40^ Presenting information that did not align with national recommendations or clinical guidelines was considered harmful.^18^ Five reviews noted the importance of safety messaging in apps.^10,33,38,43,44^ An example of incorporating a safety recommendation is an app asking the user whether they had any absolute or relative contraindications to exercise during pregnancy and information about contraindications to exercise during pregnancy.^17^

Within the major theme transparency, the following two minor themes were identified: disclosures and disclaimers. The disclosure of affiliations, ownership, sources of funding, and endorsement within apps was assessed in six reviews.^18,32,36,38,40,44^ Disclosure of affiliations notifies the user who created or developed the app. One review discussed the inclusion of disclaimers or terms and conditions and found a majority (*n* = 19/27; 70%) of apps presented users with a disclaimer or terms and conditions.^17^ Disclaimers or terms and conditions were described as absolving the app developers of liability, acknowledging user participation is at their own risk, and recognising the app is not responsible for any adverse outcomes that may occur when using the app or as a result of using the app.^17^

Within the major theme credibility, the following three minor themes were identified: recommended by a health provider, developed in consultation with a health provider, and tested or trialled. One review referred to the importance of healthcare providers’ recommendations of apps for use during pregnancy.^37^ Relatedly, two reviews referred to the importance of apps being supported in their development and dissemination by a scientific society, university, independent medical college or association, hospital, or independent body of experts or provided in partnership with health professionals.^38,40^ Two reviews assessed whether the apps included in their review had been tested or trialled through research to substantiate the apps’ quality or efficacy.^36,44^ Wu et al.^36^ found that nearly all apps containing prenatal genetic testing information (*n* = 63/64; 98%) had not been tested by research studies to determine their quality and efficacy. Of the 39 apps identified for anxiety during pregnancy, Evans et al.^44^ did not find any information provided within the apps on the development of the app, professional input, or supportive evidence.

Within the major theme privacy and security, the following minor theme was identified: data and password protection. Data protection and the use of password protection were assessed in two reviews.^33,43^ Whereas Frid et al.^43^ identified a majority (*n* = 17/29; 59%) of general pregnancy-monitoring and education apps included a privacy statement or password protection, Bachiri et al.^33^ found that only 9% (*n* = 3/33) of pregnancy-monitoring apps reviewed included at least two data protection methods and 12% (*n* = 4/33) used a password method of protection. Data protection methods mentioned include the following: protection of personally identifiable information from loss and disclosure; the use of standard physical, technical, and administrative security measures and safeguards to protect the confidentiality and security of personal information; and personal and health information that are not to be disclosed to third parties or reused for commercial purposes under any circumstances.^33^ Password protection methods mentioned included using commercially reasonable physical, managerial, and technical safeguards to preserve the integrity and security of personal information.^33^

Within the major theme app tailoring and responsiveness, the following six minor themes were identified: synchronisation with medical equipment, customisation, accessibility, tracking ability, safety alerts and notifications, and medical clearance requirements. Two reviews deemed the apps’ ability to sync with medical equipment an important feature to ensure accurate and correct recording of critical data for pregnancy monitoring.^33,40^ For example, the use of app plug-ins or other devices can assist the user to monitor foetal movement.^40^ Five reviews considered in their assessments of apps the customisation features that account for pre-existing medical conditions, tailoring of information based on a user's health status, and location-specific information to ensure the information and functionalities are tailored to the user's circumstances.^17,^^18,35–37^ Two reviews discussed the importance of accessibility when assessing apps.^36,44^ This may mean that apps are able to adapt to differences in accessibility needs (e.g. visual content and language translation) to ensure the information and functionalities are widely accessible to the target users.^35,36^ Four reviews assessed the inclusion of safety alerts and notifications within the apps reviewed.^10,33,38,40,43^ For example, apps may prompt the user to consult a doctor in case of an emergency. One review reported that nearly one-third (*n* = 3/10; 30%) of the general pregnancy-monitoring and education apps reviewed did not articulate or encourage women to contact a healthcare provider if they were concerned about decreased foetal movements or mention the risk of stillbirth.^10^ Five reviews discussed the inclusion of tracking functionality when assessing the app.^10,37,38,40,43^ Tracking functionality includes the user's ability to monitor, manage, record, and report their health-related activities in the app.^37^ For example, one review reported on apps for women with gestational diabetes with functionality for the user to monitor their blood glucose levels and record their HbA1c.^37^ One review discussed the importance of apps that promote physical activity during pregnancy, requiring medical clearance before using the app.^17^ For example, the app may ask users to confirm whether they had obtained approval or clearance from their healthcare provider.^17^

## Discussion

During pregnancy, commercially available apps have the potential to promote healthy lifestyles by improving nutrition and physical activity behaviours as well as aid in self-management of commonly diagnosed conditions.^45–47^ Yet increasingly, regulatory and safety concern issues have been raised,^10,12^ together with several seminal reviews highlighting a lack of evidence-based health information and ability to change health behaviour in apps that have been evaluated across the spectrum of pregnancy and associated conditions.^6,13–20^ In the absence of frameworks that measure safety and little synthesised evidence of app safety concerns, to the best of our knowledge, our review is the first to thematically analyse reviews of pregnancy apps and describe issues raised related to safety. Overall, we found 20 minor themes related to safety which were grouped into five major themes comprising information, transparency, credibility, privacy and security, and app tailoring and responsiveness. In the context of limited regulatory control, our results emphasise the need to increase awareness of consumers to efficiently and accurately evaluate the quality and safety of apps prior to engagement and use during pregnancy.

The availability of pregnancy-focused apps has increased in recent years in line with the prolific increase in mHealth apps more broadly. A 2021 report issued by IQVIA Institute for Human Data Science reported a doubling in the number of available mHealth apps from 2015, with over 350,000 available at time of publication.^48^ Of those, mHealth apps targeting primary care and management of specific conditions, behavioural health, fitness, and prevention were of the highest consumer demand. Previous reviews of commercially available pregnancy apps report their prolific use and popularity, reflected in total downloads as well as generally high consumer ratings on app stores for those that are most widely used.^20^ Despite this and women of reproductive age reporting their strong preferences to receive trusted health information from a variety of sources including health technologies,^49^ most recent reviews in the field report the content of mHealth apps lack rigour and evidence,^36,44^ credible expert involvement, and ability to improve behaviour on detailed evaluation, translating to increased potential for communication of harmful information to the consumer.^16,17,20,50^ This is reflected in results found here, showing low-quality scores across the majority of reviews and limited inclusion of BCTs within apps. Yet, consumer-focused research shows little concern placed on quality, credibility of information, or privacy of information by consumers when prompted, and rather functionality, technical ability, and aesthetic appeal have been reported to be of highest value to women when selecting mHealth apps.^51^ As such, there is a critical need to increase awareness in consumers when selecting mHealth apps to promote evaluation of credibility, safety, and privacy of information before engagement in a mHealth app occurs. This could be performed in conjunction with a healthcare professional, yet limited guidance exists for consumers and their healthcare professionals to efficiently evaluate app credibility. The Royal Australian College of General Practitioners developed a guiding factsheet to assist practitioners when recommending mHealth apps in the general population, emphasising quality and security concerns;^52^ however, it is unclear if an evaluation of uptake and practice modification has been conducted. Whilst research shows health professionals may be supportive of mHealth app use among their patients, barriers include lack of knowledge of effective and/or high-quality apps, liability and/or legal considerations, and limited time, all of which act to impede app promotion in healthcare settings.^16^

Widely adopted frameworks for evaluating quality of mHealth apps, including MARS,^21^ are largely designed for use in research settings, limiting their broader applicability elsewhere. MARS was also the most widely used framework to assess the quality of pregnancy apps in our review. Further, whilst MARS evaluates the domains of engagement, functionality, aesthetics, and information, critically it does not evaluate many of the themes we identified related to safety, privacy, and security, all of which are increasingly raised as major concerns in the context of minimal regulatory control for mHealth apps. Our thematic analyses identified five major themes related to safety of commercially available apps in pregnancy, including information, transparency, credibility, privacy and security, and app tailoring and responsiveness. Themes including information, transparency, and credibility relate to the evidence base of information within the mHealth app, the use of high-quality referencing, including clinical guideline documents and engagement with relevant health professional expertise in development of the mHealth app. Increased rigour in content development reduces likelihood of inaccurate or harmful information. An included review of apps to monitor foetal movements reported inaccurate information in all apps reviewed, including expected number of kicks per hour, methods to increase kicks, and use of Doppler devices to monitor foetal wellbeing.^10^ Importantly, several apps reviewed neglected to encourage partnership with a healthcare professional if the user was concerned or if decreased movements were experienced by the user.^10^

Healthcare providers' input at the app development stage would increase rigour of information and potential endorsement of an app’s use once launched by relevant healthcare organisations, networks, or governing bodies, increasing credibility and reassurance for consumers when selecting apps to engage with during pregnancy. However, this is complex, requiring consideration of, as well as tailoring for, contextual factors, including local or national clinical practices guidelines for management of different conditions during pregnancy that are highly variable between countries,^18,37^ as noted in the major theme of app tailoring and responsiveness. Tailoring of mHealth apps is of importance in the context of consumer experience including language preferences and health and digital health literacy all of which are highly variable. A 2021 review of apps containing prenatal genetic testing information found 95% of apps reviewed had a readability level higher than the acceptable threshold for general population use and 92% had no visual information to improve ease of understanding, including videos, diagrams, or tables.^36^ This has significant accessibility implications particularly for culturally and linguistically diverse and/or non-English-speaking consumers, who have lower app uptake compared with English-speaking consumers, potentially due to a lack of availability of multilingual apps.^6^ Presentation of highly complex information should be equitable to all consumers and could be enhanced by co-development strategies. This includes consumer involvement in the app development phase to evaluate readability, cultural sensitivity, relevancy, and acceptability of digital content. Finally, privacy and security of mHealth apps including protection of data disclosure and authentication measures for accessing personal information was emphasised across three reviews.^33,36,43^ One included review of mobile personal health records reported under 50% contained a privacy policy and just 9% and 12% of apps storing personal information contained at least two data protection methods and had password protection, respectively.^33^ Broader evaluation of over 20,000 mHealth apps in 2021 reported ~95% of the top 556 most popular health apps contained a privacy policy, which progressively reduced with diminishing popularity,^53^ with 28% of apps overall found to contain no privacy policy. Concerningly, 88% of apps had potential to collect user information via back-end codes, and 25% of apps shared user information that contravened their listed policy. Despite this, just 1% of consumer reviews of the apps cited privacy concerns, potentially indicating a lack of awareness and/or concern about potential risk of data disclosure or transmission.^53^

Ultimately, currently little regulatory control exists to ensure quality and safety of mHealth apps available to women during pregnancy. In the USA, the Food and Drug Administration has provided guidelines that mandate the regulation of apps.^54^ However, currently this only includes regulation of apps that either act as a medical device or control a medical device, and, as such, mHealth apps that fall outside of this function are considered to be of less harm to public safety and thus have no regulatory oversight. Similar regulation exists in other developed countries including the Therapeutic Goods Association in Australia.^55^ In 2022, the UK Government published a voluntary code of practice for developers to improve security of apps available in the UK, including processes related to reporting of software vulnerabilities to developers and publishing security and privacy information to consumers that can be easily understood.^56^ Despite increasing awareness of the need to place regulatory guidance on app developers for the benefit of the broader population using mHealth apps, currently, consumers are ultimately responsible for making informed decisions around engagement, use, and adoption of information. In the context of mHealth apps targeting pregnancy, it is, therefore, critical that consumers understand the credibility of content and advice, the limitations of an app and when engagement with a healthcare professional is necessary. Evaluating how their privacy is maintained when disclosing and storing personal information is also essential. This could include decision-making tools to guide engagement with apps, emphasising the need for more consumer-based co-design research in this area. As suggested previously, streamlined and transparent pathways for consumers to report safety concerns are also warranted to enable greater awareness of potentially harmful apps.^16^

### Strengths and limitations

We note several limitations. First, the primary focus of our review included evaluating commercially available apps during pregnancy to scope current issues related to safety concerns. We, therefore, included any type of app review, including content analyses, content evaluations, and systematic or scoping reviews. We defined ‘reviews’ as a study that assessed at least two commercially available apps. We did not include reviews evaluating efficacy of apps on health outcomes during pregnancy and associated safety. Second, due to the constantly evolving nature of app development, it is possible that apps assessed in the included reviews discussed here may now be outdated or unavailable. Additionally, we chose to limit our search to only include literature published between January 2011 and April 2022 due to this constantly evolving nature of app development to increase the likelihood of identifying reviews inclusive of currently available commercial apps. However, limiting our search in this way may have potentially excluded relevant reviews. We included literature written in English, and, therefore, it is possible we have omitted reviews published in other languages. Third, as a definition of safety and security for pregnancy apps does not exist, we were informed by several seminal reviews of mHealth apps^17,^^27–30^ that capture safety and security components to inform the formulation of codes and data extraction as well as a rigorous thematic analysis approach to reduce potential methodological bias. Moving forward, the definitional components set out in this review form the foundational understanding of safety for pregnancy-specific apps but need to be validated by the target population. Other strengths of the present review include focus on commercially available apps that have much higher penetration compared with apps that are tested for efficacy in controlled environments. We applied methodological rigour in our developed search strategy; included a broad evidence base comprising systematic reviews, scoping reviews, and literature reviews; and used two reviewers for the thematic analysis process.

## Conclusion

mHealth apps during pregnancy are common and an important source of information for women across the spectrum of healthy lifestyles and condition management. Our comprehensive thematic analysis of 16 reviews, comprising a total of 445 of mHealth apps in pregnancy, highlights the significant safety concerns that exist in the context of suboptimal app quality and their ability to influence behaviour. Information, transparency, credibility, privacy and security, and app tailoring and responsiveness were frequently reported as major concerns related to commercially available apps. In the context of limited regulatory control, our results emphasise the need to increase awareness of consumers and healthcare providers to efficiently evaluate the quality and safety of apps prior to engagement and use during pregnancy.

## Supplemental Material

sj-docx-1-dhj-10.1177_20552076231198683 - Supplemental material for Safety considerations for assessing the quality of apps used during pregnancy: A scoping review Click here for additional data file. Supplemental material, sj-docx-1-dhj-10.1177_20552076231198683 for Safety considerations for assessing the quality of apps used during pregnancy: A scoping review by Alayna Carrandi, Melanie Hayman and Cheryce L Harrison in DIGITAL HEALTH

sj-docx-2-dhj-10.1177_20552076231198683 - Supplemental material for Safety considerations for assessing the quality of apps used during pregnancy: A scoping review Click here for additional data file. Supplemental material, sj-docx-2-dhj-10.1177_20552076231198683 for Safety considerations for assessing the quality of apps used during pregnancy: A scoping review by Alayna Carrandi, Melanie Hayman and Cheryce L Harrison in DIGITAL HEALTH

sj-docx-3-dhj-10.1177_20552076231198683 - Supplemental material for Safety considerations for assessing the quality of apps used during pregnancy: A scoping review Click here for additional data file. Supplemental material, sj-docx-3-dhj-10.1177_20552076231198683 for Safety considerations for assessing the quality of apps used during pregnancy: A scoping review by Alayna Carrandi, Melanie Hayman and Cheryce L Harrison in DIGITAL HEALTH
