# Supplementary material for: Safety considerations for assessing the quality of apps used during pregnancy: A scoping review
Source: Digit Health. 2023 Sep 4;9:20552076231198683. doi: 10.1177/20552076231198683 (PMC10478559; doi:10.1177/20552076231198683)
Supplement: sj-docx-2-dhj-10.1177_20552076231198683 - Supplemental material for Safety considerations for assessing the quality of apps used during pregnancy: A scoping review [file sj-docx-2-dhj-10.1177_20552076231198683.docx]

**Apendix 2.** Full search strategy in accordance with PRISMA 2020 reporting requirements.

| **Database platform/interface (Title of the database searched)** | **Database date range** | **Date database last searched or consulted** | **Years covered by the search and other filters** | **Complete search strategy** | **Total results from search** |
| --- | --- | --- | --- | --- | --- |
| Pubmed | 1963 to present | 28^th^ April 2022 | January 2011 – April 2022; English | #1 (mhapps[Title/Abstract] OR mobile app*[Title/Abstract] OR app[Title/Abstract] OR apps[Title/Abstract] OR phone application[Title/Abstract] OR health application[Title/Abstract] OR iphone*[Title/Abstract] OR smartphone*[Title/Abstract] OR android*[Title/Abstract] OR smart phone*[Title/Abstract] OR ipad[Title/Abstract] OR ipads[Title/Abstract] OR tablet[Title/Abstract]) OR ("mobile applications"[MeSH Major Topic] OR "smartphone"[MeSH Major Topic])  #2 (Content analysis[Title/Abstract] OR systematic review of systematic reviews[Title/Abstract] OR systematic review[Title/Abstract] OR review[Title/Abstract] OR evaluation[Title/Abstract] OR scoping review[Title/Abstract] OR content evaluation[Title/Abstract] OR mobile usability evaluation[Title/Abstract] OR appraisal[Title/Abstract]) OR ("systematic reviews as topic"[MeSH Major Topic])  #3 ("pregnant women"[MeSH Major Topic] OR "pregnancy"[MeSH Major Topic] OR "maternal health"[MeSH Major Topic] OR "maternal health services"[MeSH Major Topic] OR "prenatal care"[MeSH Major Topic] OR "perinatal care"[MeSH Major Topic]) OR (Pregnan*[Title/Abstract] OR matern*[Title/Abstract] OR gestation*[Title/Abstract] OR antenatal[Title/Abstract] OR ante-natal[Title/Abstract] OR prenatal[Title/Abstract] OR pre-natal[Title/Abstract] OR perinatal[Title/Abstract] OR peri-natal[Title/Abstract])  #1 and #2 and #3 | 405 |
| Elsevier (Scopus) | 1957 to present | 28^th^ April 2022 | January 2011 – April 2022 | ( TITLE-ABS-KEY ( mhapps OR "mobile app*" OR app OR apps OR "phone application" OR "health application" OR "iphone*" OR "smartphone*" OR "android*" OR "smart phone*" OR "ipad" OR "pads" OR "tablet" ) AND TITLE-ABS-KEY ( content AND analysis OR "systematic review off systematic reviews" OR systematic AND review OR systematic AND search OR scoping AND review OR content AND evaluation OR "mobile usability evaluation" OR appraisal ) AND TITLE-ABS-KEY ( pregnan* OR matern* OR gestation* OR antenatal OR ante-natal OR prenatal OR pre-natal OR perinatal OR peri-natal ) ) | 3 |
| Ebsco host (CINAHL Plus) | 1957 to present | 28^th^ April 2022 | January 2011 – April 2022; English | S1  TI ( mhapps OR “mobile app*” OR app OR apps OR “phone application” OR “health application” OR iphone* OR smartphone* OR android* OR smart phone* OR ipad OR ipads OR tablet) OR AB (mhapps OR “mobile app*” OR app OR apps OR “phone application” OR “health application” OR iphone* OR smartphone* OR android* OR smart phone* OR ipad OR ipads OR tablet) OR MH "Mobile applications" OR MH Smartphone  S2 TI ( Content analysis OR “systematic review of systematic reviews” OR systematic review OR systematic search OR scoping review OR content evaluation OR “mobile usability evaluation” OR appraisal ) OR AB ( Content analysis OR “systematic review of systematic reviews” OR systematic review OR systematic search OR scoping review OR content evaluation OR “mobile usability evaluation” OR appraisal ) OR MH "Systematic review"  S3 TI ( Pregnan* OR matern* OR gestation* OR antenatal OR ante-natal OR prenatal OR pre-natal OR perinatal OR peri-natal ) OR AB ( Pregnan* OR matern* OR gestation* OR antenatal OR ante-natal OR prenatal OR pre-natal OR perinatal OR peri-natal ) OR ( (MH "Pregnancy") OR (MH "Expectant Mothers") OR (MH "Maternal Health Services") OR MH "Prenatal Care" OR MH "Perinatal Care"  S4  S1 and S2 and S3 | 94 |
| Ovid (Medline ® and Epub) | 1946 to present | 28^th^ April 2022 | January 2011 – April 2022 | 1 Mobile applications/ OR smartphone/  2 (mhapps OR mobile app* OR app OR apps OR phone application OR health application OR iphone* OR smartphone* OR android* OR smart phone* OR ipad OR ipads OR tablet).mp.  3 Systematic review/  4 (Content analysis OR systematic review of systematic reviews OR systematic review OR systematic search OR scoping review OR content evaluation OR mobile usability evaluation OR appraisal).mp.  5 Exp Pregnancy/ or pregnant women/ OR maternal health services/ OR maternal health/ OR prenatal care/ OR perinatal care/  6 (Pregnan* OR matern* OR gestation* OR antenatal OR ante-natal OR prenatal OR pre-natal OR perinatal OR peri-natal).mp.  7 1 or 2  8 3 or 4  9 5 or 6  10 7 and 8 and 9 |  |
| Ovid (Cochrane database of systematic reviews) | 2005 to present | 28^th^ April 2022 | January 2011 – April 2022 | 1 (mhapps or "mobile NEXT app*" or app or apps or "phone application" or "health application" or "iphone*" or "smartphone*" or "android*" or "smart phone*" or "ipad" or "ipads" or "tablet").mp.  2 (Pregnan* or matern* or gestation* or antenatal or ante-natal or prenatal or pre-natal or perinatal or peri-natal).mp.  3 ("Content analysis" or "systematic review of systematic reviews" or "systematic review" or "systematic search" or "scoping review" or "content evaluation" or "mobile usability evaluation" or "appraisal").mp.  4 1 and 2 and 3 | 156 |
| Web of science (Web of Science Core Collection, Biological Abstracts, BIOSIS Previews, Current Contents Connect, Data Citation Index, KCI-Korean Journal Database, Russian Science Citation Index, SciELO Citation Index) | 1900 to present | 28^th^ April 2022 | January 2011 – April 2022 | #1  TI=(mhapps OR "mobile app*" OR app OR apps OR "phone application" OR "health application" OR "iphone*" OR "smartphone*" OR "android*" OR "smart phone*" OR "ipad" OR "ipads" OR "tablet")  OR  AB=(mhapps OR "mobile app*" OR app OR apps OR "phone application" OR "health application")  #2  TI=(Content analysis OR “systematic review of systematic reviews" OR systematic review OR systematic search OR scoping review OR content evaluation OR "mobile usability evaluation" OR appraisal)  OR  AB=(Content analysis OR “systematic review of systematic reviews" OR systematic review OR systematic search OR scoping review OR content evaluation OR "mobile usability evaluation" OR appraisal)  OR TS=("Systematic review")  #3  TS=(Pregnancy or pregnant women OR maternal health OR maternal health services OR prenatal care OR perinatal care)  OR  TI=(Pregnan* OR matern* OR gestation* OR antenatal OR ante-natal OR prenatal OR pre-natal OR perinatal OR peri-natal)  OR  AB=(Pregnan* OR matern* OR gestation* OR antenatal OR ante-natal OR prenatal OR pre-natal OR perinatal OR peri-natal)  #4  #3 AND #2 AND #1 | 251 |
