# Supplementary material for: Safety considerations for assessing the quality of apps used during pregnancy: A scoping review
Source: Digit Health. 2023 Sep 4;9:20552076231198683. doi: 10.1177/20552076231198683 (PMC10478559; doi:10.1177/20552076231198683)
Supplement: sj-docx-3-dhj-10.1177_20552076231198683 - Supplemental material for Safety considerations for assessing the quality of apps used during pregnancy: A scoping review [file sj-docx-3-dhj-10.1177_20552076231198683.docx]

**Appendix 3.** Complete data extraction form.

| Basic study characteristics | |
| --- | --- |
| Author |  |
| Title |  |
| Year of Publication |  |
| Health domain within pregnancy (e.g., physical activity, GDM, etc.) |  |
| Aim of the review |  |
| Methods | |
| Database / Platform/s searched |  |
| Date range of search |  |
| Inclusion/ exclusion criteria (e.g., where apps were sourced from, paid or free apps, etc.) |  |
| Evaluation tools | |
| Evaluation tool #1 | |
| Name of tool |  |
| Is the tool validated? (y/n) |  |
| Scoring of the tool if modified from a published tool or author created tool |  |
| Thematic extraction of anything related to safety over and above what is included in the tool |  |
| How many people extracted the data? |  |
| Evaluation tool #2 | |
| Name of tool |  |
| Is the tool validated? (Y/N) |  |
| Scoring of the tool if modified from a published tool or author created tool |  |
| Thematic extraction of anything related to safety over and above what is included in the tool |  |
| How many people extracted the data? |  |
| Evaluation tool #3 | |
| Name of tool |  |
| Is the tool validated? (Y/N) |  |
| Scoring of the tool if modified from a published tool or author created tool |  |
| Thematic extraction of anything related to safety over and above what is included in the tool |  |
| How many people extracted the data? |  |
| Results | |
| Number of apps included |  |
| Score of the framework 1 (related to evaluation tools) |  |
| Score of the framework 2 (related to evaluation tools) |  |
| Score of the framework 3 (related to evaluation tools) |  |
| Results related to safety (from thematic extraction of safety or quantitative)) |  |
| Main limitations |  |
| Discussion | |
| Does the discussion mention themes directly relate to safety? (Y/N) |  |
| Excerpt of safety-related information |  |
